# On Metro-Peritonitis, Following the Use of the Ordinary Female Syringe: A Plea for the Vaginal Irrigator

**Published:** 1875-05-01

**Authors:** Thomas More Madden


### ON METRO-PERITONITIS, FOLLOWING THE USE OF THE ORDINARY FEMALE SYRINGE: A PLEA FOR THE VAGINAL IRRIGATOR.

READ BEFORE THE OBSTETRICAL SOCIETY OF DUBLIN, FEBRUARY 13TH, 1875,
BY THOMAS MORE MADDEN, M.D., M. R. I. A.

*From the Obstetrical Journal of Great Britain and Ireland, April*, 1875.

THE use of the vaginal syringe is the most common, and, until recently, was almost the only local treatment resorted to in uterine disorders. As this instrument is now so generally employed in all cases of real or suspected disease of the womb, that is to say, in nine-tenths of the complaints peculiar to women, and as it is freely ordered by medical men and habitually used by patients without any special caution or apprehension of possible danger, the following case appears to me not unworthy of the consideration of this Society:

Mrs. W., a healthy young lady, aged twenty-four, was delivered on the 16th of July, 1874, of her first child. The labor, which was very tedious in the first stage, was rendered difficult by rigidity of the os uteri and malposition of the child's head, and had to be completed with the assistance of my long forceps, an instrument of a peculiar construction which I have elsewhere described.* Her convalescence presented nothing remarkable. On the 24th of August she called on me, complaining of pain in the back, a profuse yellow leucorrheal discharge, and a distressing bearing-down sensation, which prevented her from walking, or even standing with any comfort. The vagina was congested, the os patulous, and the uterus slightly prolapsed and enlarged. Absolute rest was enjoined, a tonic prescribed, and she was directed to use an astringent vaginal injection.

A few nights afterwards I was sent for, but being out of town, Dr. Egan, of Talbot street, saw her, and found her suffering from intense uterine colic, which had come on during the use of the injection when going to bed a couple of hours previously. She was then cold, almost pulseless, and apparently semi-moribund. Having promptly administered stimulants and applied sinapisms over the heart, etc., Dr. Egan, as he subsequently informed me, considered her condition so precarious, that he called in the assistance of Dr. Johnston, in consultation; and owing to the efficacious measures adopted, the most urgent symptoms were abated, the warmth was restored to the extremities, the pulse became apparent at the wrist, and the severe uterine pain was for the time considerably relieved.

On the following morning, I visited her with Dr. Egan, who kindly left the case in my care, and found her still in a state of extreme prostration, and again suffering from frequent paroxysms of violent uterine pain and almost continual retching, with great tenderness over the abdomen, and especially over the uterus, which was as large and hard as it should be immediately after delivery. Her pulse was 140, and so weak that it could hardly be counted; her respiration was sighing; countenance pale and anxious, and the skin cold and clammy.

Poultices and anodyne stupes were applied to the abdomen; hydrocyanic acid draughts were given to allay the retching, and her strength was supported by enemata of brandy and beef-tea, with small doses of liquor opii. I again saw her the same evening; the incessant sickness and uter-

* Vide *Lancet*, June 20th, 1874.

ine pain continued; she was suffering from constant thirst, but no sooner did she take any fluid, or even a piece of ice, than it was immediately rejected. She was ordered therefore to take nothing whatever by the mouth. A drop of hydrocyanic acid was added to each enema, and the expedient (which I have not seen alluded to by any writer) certainly diminished, though it did not stop the vomiting. Notwithstanding the great depression of the pulse, the local uterine inflammation was so marked, that I applied a few leeches over the seat of pain, and this gave considerable relief to the suffering, whilst the pulse actually became feebler and less compressible than before their application. Besides this, the solution of muriate of morphia was used hypodermically to relieve pain and calm the nervous system, though no sleep was induced by it.

On the 31st, the metro-peritoneal pain had in great measure subsided, but there was yet considerable tenderness over the uterus, which continued to be perceptibly tumefied. Her pulse was 140 in the minute, weak and compressible; tongue dry and furred; decubitus dorsal, and sunk down to the back of the bed, and she was still suffering much from the constant nausea and dry retching. The same treatment was pursued as on the previous day.

*September 1st.*—She had slept a little last night; her pulse had fallen to 120, and the uterine tenderness was much less than yesterday; the retching and inability to take any nourishment or medicine by the mouth, though less marked, still continued. I suggested a consultation. Dr. McClintock accordingly saw her with me that afternoon, and I had the satisfaction of having my view of the case confirmed by this eminent gynecologist, who, in addition to the treatment already referred to, advised oxalate of cerium, in small doses, every third hour, and Dr. Halahan's egg drink to check the retching.

*September 2d.*—The egg drink, in teaspoonful doses, had agreed with her, and the oxalate of cerium pills, which at first had been rejected, were now retained; it was necessary, however, to continue the enemata, etc., as before, though she gradually improved each day until the 5th, when she was able to take a little iced chicken jelly by the mouth, and from this time her recovery was rapid, so that on the 11th she was able to sit up for a couple of hours, and was soon convalescent.

In the foregoing case, the uterine colic and subsequent metro-peritonitis followed so immediately on the use of the vaginal syringe as to leave no room for any doubt of their being caused by the fluid having been injected through the patulous os into the uterus, and probably also by some of it having passed through an enlarged or dilated Fallopian tube into the peritoneal cavity.

The probability of an accident of this kind attending the use of the ordinary female syringe is practically ignored by the great majority of gynecologists; yet cases similar to mine, although somewhat rare, have been recorded by other practitioners; but they would appear to have made less impression on the minds of those engaged in this branch of medicine than the importance of the subject demands.

Dr. Tilt says: "Only once have I been led to believe that the patient injected some portion of the fluid into the cervical canal. A lady was suffering from chronic uterine inflammation; the womb was low and slightly retroflected; the os uteri patulous; and after injecting a solution of acetate of lead, as the patient thought, in the usual way, she was suddenly seized with severe uterine pains, rigors and intense cold. She got better when in bed, by means of abdominal poultices and hot drinks, and no bad consequences followed this attack." *

Dr. J. H. Bennet says: "When disease really exists in the uterine

* Dr. Tilt's "Handbook of Uterine Therapeutics." Third Edition. P. 54. London, 1868.

cavity, the injections would, no doubt, do much good, and were they safe, would be preferable to the solid nitrate of silver applied with the porte-caustic; but there is reason to believe that uterine injections are not safe, and I do not now resort to them. Several deaths occurred in Paris during my residence there, from metro-peritonitis, brought on by their use. One took place in the female ward of M. Jobert, in the Hospital St. Louis, and under my care, as I was then his house-surgeon. The patient, a fine healthy young woman of twenty-four, was afflicted with a large fibrous tumor of the uterus, which had much developed that organ, and had, no doubt, opened the os internum. M. Jobert was at that time trying the effects of the so-called uterine injections, and injected one astringent injection into the cervical canal of this young female, there being a slight muco-purulent discharge from the os. Shortly after she was seized with rigors, fever, and severe abdominal pain, and in a few days died of peritonitis. I performed the post-mortem and found nothing but the lesions of peritonitis, and the ovarian tumor embedded in a womb developed to the size which it presents in the fourth month of pregnancy. The fluid of the injection must have penetrated freely into the uterus, through the open os, and thence have passed along the Fallopian tube into the cavity of the peritoneum, thus causing fatal peritonitis." †

M. Bernutz relates the history of a case of peritonitis "which resulted immediately from the administration of a cold vaginal douche." ‡

These three quotations are the only references that I have met with to the dangers which may follow the use of the ordinary vaginal syringe, and the paucity of similar observations is certainly a fair argument that the accident in question is by no means frequent. Still the mere possibility of such serious, or even fatal results, being thus produced, should, I think, render gynecologists more cautious than is generally the case in their recommendation of this almost universally-employed and much-abused instrument. For my own part, I have been long convinced of the inconvenience of the vaginal syringe in ordinary use, as well as of the possible ill effects of its misapplication. Some years ago I pointed out in this place how uncertain and undue might be the force with which injections can be thus thrown into the vagina or into the uterus, and I endeavored to show that, even when not positively injurious, this instrument must necessarily be imperfect in its action, and inconvenient in its use. To produce any permanent beneficial effect by injections, in a case of congestion or inflammation of the cervix uteri for instance, the injected fluid must be kept in contact with the inflamed or congested part for a certain fixed length of time, and at a certain unvarying degree of heat, without any sudden alternations in either its temperature or the force with which it is impelled. None of these intentions can be carried out when the common siphon syringe is made use of, as the fatigue of working that instrument, and the position of the patient, which is so irksome during its employment. effectually prevent its being used more than a few minutes continuously. Moreover, the injected fluid is sent by it into the vagina in irregular intermitting jets, the force of which is directed against the inflamed part with a degree of violence that may be injurious, or of weakness that may be utterly ineffective, and which is regulated by the strength or patience of the operator, rather than by the necessities of the case. In order to obviate these difficulties, various forms of utero-vaginal syringes, douches, and irrigators have been devised. It may be in the recollection of some present, that a couple of years ago, I exhibited a very simple,

† "On Inflammation of the Uterus and its Appendages." By J. H. Bennet, M.D. Fourth Edition. P. 146. London, 1861.

"Clinical Memoirs on the Diseases of Women." By MM. Bernutz and Goupil. Translated by Dr. Meadows. Vol. II. P. 78. London, 1868.

and, I believe, very effective instrument, for the same purpose before the Society. The irrrigator in question* (a description and drawing of which may be seen in the second volume of our proceedings, p. 183), is one I have now used for some years, and which, without asserting that it is in any way superior to other instruments of the same kind, I have found more generally useful than the ordinary syringe. With this irrigator, the accident which forms the subject of the present paper, could not have occurred, as it is merely a siphon, so constructed that it can be set in action, or stopped in an instant at the will of the patient.

It has, moreover, the great advantage of being very portable; may be readily used whenever a vessel of water can be obtained; is capable of sending a gentle continuous current of water, plain or medicated, and at any temperature, into the vagina, or even into the uterine cavity itself, if ever that measure—rarely required in gynecological, as distinguished from obstetric practice—should be considered expedient, and this, too, in any position, sitting or recumbent, and for any length of time that may be advisable, without causing the slightest fatigue to the patient. The advantages of the irrigator over the common syringe, I have proved by experience during the last few years, and the comfort and benefit which those patients who have employed it have derived from its use, would lead me to recommend any gynecologist who has not hitherto done so, to give a trial to the irrigator as a substitute for the vaginal syringe in most cases. This instrument is not only effective and easily used, but is also easily constructed. Dr. Graily Hewitt, Dr. Kidd, and several others, have recommended and devised irrigators which many would, perhaps, prefer to the one I have brought before this Society. But it is in the power of anyone to convert an ordinary vaginal syringe into an excellent irrigator by the mere addition of two pieces of india rubber tubing and a small stopcock to it. These may be attached to any syringe, which may be thus made, perhaps fully as effectual as the most complicated instrument that could be devised for the purpose.

In another point of view, cases such as those just referred to, appear to me of some interest, namely: as to their bearing on the recent discussion as to the safety of strong astringent injections into the uterus immediately after delivery, in order to arrest hæmorrhage. Against this practice, it has been urged that there is danger of forcing the injected fluid through the open uterine sinuses into the circulation, or through the Fallopian tubes into the peritoneal cavity, thus causing, in the former case, death from embolism, and in the latter giving rise to fatal peritonitis. As on a former occasion, when this subject was under the consideration of the Obstetrical Society, I expressed a strong opinion against the probability of such an accident being thus produced, I feel bound to say that the case I have now related has, to some extent, modified my views on this point. For if metro-peritonitis may be occasioned, as it was in this instance, by a fluid injected into the uterus five weeks after delivery, it is obviously much more probable that a similar effect might be thus occasioned immediately after parturition, when the uterine vessels and passages are so enlarged and pervious. But I should also add that the possibility of such an accident does not in the least alter my opinion of the utility of the powerful styptic brought into midwifery practice by Dr. Barnes, nor would it deter me from again resorting to the injection of the solution of perchloride of iron in any case of post-partum hæmorrhage which could not be otherwise controlled.

The President observed that Dr. Madden's brief paper opened up a very important subject for discussion. He did not think the occurrence of uterine colic following the injection of fluids by the syringe, was a very rare occurrence, inasmuch as he had seen

* Made by Whyte, Upper Sackville Street, Dublin.

three cases of it in his own practice. In one case only a few drops of glycerine were injected into the cavity of the uterus, as recommended by Dr. Marion Sims, and it produced most intense colic, but no peritonitis or endo-metritis followed. Some two years ago he was called, late at night, to see a patient whom he had directed to use a weak solution of borax injected into the vagina with an ordinary syringe. He found her in a state of collapse, suffering from pain referred to the uterus, and sickness of stomach. Her symptoms were speedily relieved, and no inflammation followed; while a less severe attack occurred in a patient who used tepid water only. He thought these cases in which the injection of a fluid into the uterus was followed by colic, were far from being of very rare occurrence, and he advised that the central hole in the nozzle of the syringe be stopped, as a means of preventing this accident. He did not think, however, that the data given by Dr. Madden carried out his theory that the fluid passed into the Fallopian tubes, and thence into the peritoneum. The phenomena in Dr. Madden's case might be explained by the occurrence of a severe attack of endo-metritis in the first instance, followed by peritonitis. The exact same train of symptoms which Dr. Madden had described—the prostration, collapse, and vomiting—occurred in a patient where he (the President) had swabbed out the uterus with perchloride of iron. The patient was suffering from profuse hæmorrhage occurring some weeks after abortion; the os was patulous, and he had no difficulty in passing a pledget of cotton, saturated with the styptic, into the uterus; this was followed by a train of symptoms exactly similar to those Dr. Madden had described, but it was impossible that the fluid was passed through the Fallopian tubes. Certainly Dr. Madden was quite right in saying that vaginal injections were not perfectly free from danger. He (the President) greatly preferred a douche, similar to that spoken of by Dr. Madden, to the use of a vaginal syringe, and he had recently (acting on the suggestion of Dr. Emmet, of New York,) carried out that plan extensively. Dr. Emmet advocated strongly this vaginal irrigation with water, varying from 95° to 105° of temperature. He based his treatment on the analogy that existed between the effects produced by it and an ordinary linseed poultice, or other hot application, applied to the skin. He says: If you leave a linseed poultice on for some time, the skin becomes corrugated and white, showing that, though the first effect of the warmth was to attract the blood to the surface, this was followed by contraction and diminution in size of the calibre of the blood-vessels. He (the President) did not think this analogy held good, for, in the case of the application of a poultice four or five hours elapsed before bloodlessness of the part occurred, while the vaginal douche could hardly be prolonged over fifteen or twenty minutes. Therefore, although considerable benefit followed in many cases from the hot vaginal douche, he did not think the theory was quite correct. To use the vaginal douche efficiently, it was necessary that the hips be raised higher than the shoulders, so that the vagina may be distended with the fluid; that the stream of fluid should be continuous, and that it should be kept up to an equal temperature. He (the President) had tested this method extensively. In a case of metritis, where the temperature of the water was raised to 110°, the patient derived much benefit. In another, a case of vaginitis, the patient had been subjected previously to treatment of various kinds without benefit. Two gallons of water, temperature 105°, were used twice daily. She derived the greatest relief, and was, in fact, cured by it. The great difficulty in carrying out this treatment, where a large quantity of water necessarily is used, was that the bed-pan on which the patient lay had to be emptied constantly. To obviate that difficulty he had introduced the plan of attaching a tube to a bed-pan of peculiar shape, by which means the water was carried away. The pan must be placed on a firm surface, and, if this precaution is taken, no trouble whatever is experienced in using it. The water for the douche should be contained in a case such as that used by surgeons for irrigation, and be raised to the height of a few feet above the level of the couch or table on which the patient rests.

---

HETEROPLASTY.—M. B. Anger has communicated to the Academy of Sciences of Paris a paper wherein he mentions that he has succeeded in transplanting large pieces of skin taken after recent amputations, upon large ulcers and extensive losses of substance after burns. This he calls hetoroplasty. The author uses the whole thickness of the skin, and takes care that the temperature of the part to be transplanted be not low. In fact he has laid patients side by side so as to convey the skin of one without the least delay upon the ulcer of the other. M. Anger is very careful to say that the general health of those patients who furnish skin should be thoroughly investigated before the operation.—*London Lancet.*

